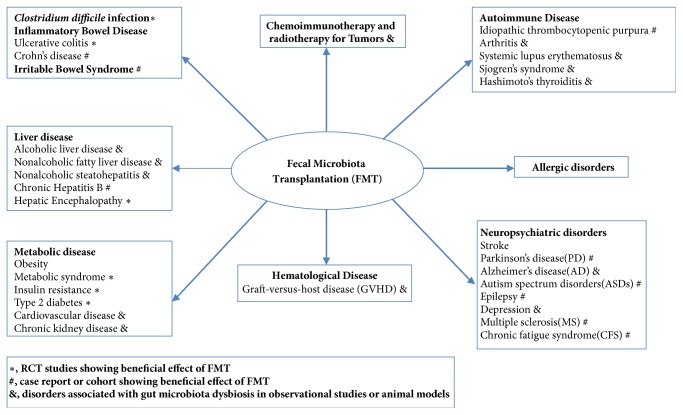# Are There Potential Applications of Fecal Microbiota Transplantation beyond Intestinal Disorders?

**DOI:** 10.1155/2019/3469754

**Published:** 2019-07-29

**Authors:** Youlian Zhou, Haoming Xu, Hongli Huang, Yingfei Li, Huiting Chen, Jie He, Yanlei Du, Ye Chen, Yongjian Zhou, Yuqiang Nie

**Affiliations:** ^1^Department of Gastroenterology, Guangzhou Digestive Disease Center, Guangzhou First People's Hospital, School of Medicine, South China University of Technology, Guangzhou 510180, China; ^2^Department of Gastroenterology, Guangzhou Digestive Disease Center, Guangzhou First People's Hospital, Guangzhou Medical University, Guangzhou 510180, China; ^3^State Key Laboratory of Organ Failure Research, Guangdong Provincial Key Laboratory of Gastroenterology, Department of Gastroenterology, Nanfang Hospital, Southern Medical University, Guangzhou, 510515, China

## Abstract

Intestinal microbial dysbiosis is associated with various intestinal and extraintestinal disorders. Fecal microbiota transplantation (FMT), a type of fecal bacteriotherapy, is considered an effective therapeutic option for recurrent* Clostridium difficile* infection (rCDI) and also has important value in other intestinal diseases including irritable bowel syndrome (IBS) and inflammatory bowel disease (IBD). The purpose of this review is to discuss promising therapeutic value in extraintestinal diseases associated with gut microbial dysbiosis, including liver, metabolic, chronic kidney, neuropsychiatric, allergic, autoimmune, and hematological diseases as well as tumors.

## 1. Introduction

The gut microbiota is an “invisible organ” of the human body important for health. There are diverse microbes in different anatomical areas of the gut, throughout the proximal to distal gastrointestinal (GI) tract. The large intestine harbors the majority of the gut's flora [1]. In addition to differences in the geographical distribution of gut microbiota, dynamic microbial population also develops with age, with rapid changes until 2 to 3 years of age, when adult-like gut microbiota composition and stability are established [2, 3]. Firmicutes, Proteobacteria, and Bacteroidetes are the most abundant phyla, together accounting for up to 95% of the sequences, while Fusobacteria, Actinobacteria, Tenericutes, Verrucomicrobia, Synergistetes, and Cyanobacteria each account for 0.1%-5% of the sequences in a healthy adult [4, 5].

Microbiota plays a variety of roles and has various functions in the gut [6]. In addition to breaking down foods and synthesizing nutrients, microbiota plays an important role in the immune system [7–9], provides colonization resistance [10, 11], protects against epithelial injury [12], promotes both angiogenesis [13, 14] and fat storage [15], modulates human bone mass density [16], modifies the nervous system [17], and metabolizes therapeutic agents into active compounds [18].

Gut microbiota homeostasis can be disrupted by many factors, including medications, diet, disease states, and vaccination [1]. Previous research suggested that gut microbial alterations are associated with many intestinal disorders and various extraintestinal disorders such as obesity, metabolic dysfunction [19–21], neuropsychiatric conditions [22], autoimmune diseases [23], and tumors [24]. Targeting the gut microbiota is being considered as an option to improve human health. Fecal microbiota transplantation (FMT), which transfers fecal microbiota from healthy donors to restore the gut microbiota of a diseased individual [25–27], has attracted great interest in recent years and has been occasionally used to treat* Clostridium difficile *infection (CDI) with great success [28]. In this brief review, we will summarize the relationship between gut microbiota and inter- or extraintestinal disorders, and current clinical use or emerging applications of FMT in recent years (Figure 1).

## 2. FMT for Intestinal Disorders

### 2.1. Clostridium Difficile Infection (CDI)

CDI is a common cause of antibiotic associated with diarrhea, and its pathology is mediated by toxins secreted by bacteria [29]. Increasing evidence, including meta-analyses, systematic reviews, and randomized controlled trials (RCTs), has confirmed that FMT is effective for the treatment of recurrent* Clostridium difficile* infection (rCDI) [30–33]. According to the 2016 European consensus conference on FMT in clinical practice, FMT is considered as a therapeutic option for both mild and severe rCDI (quality of evidence: high. Strength of recommendation: strong), and it can also be considered as a treatment option for refractory CDI (quality of evidence: low. Strength of recommendation: strong). However, there is not enough evidence emphasizing that it can be used as a single therapy for CDI (quality of evidence: low. Strength of recommendation: weak) [34]. In one randomized trial investigating the effectiveness of FMT in rCDI patients using microbiological and/or clinical resolution, a combination of FMT and vancomycin was found to be superior to a treatment regimen of vancomycin or fidaxomicin [35].

### 2.2. Inflammatory Bowel Disease (IBD)

Although IBD etiology and pathogenesis are unclear, genetic links to host pathways suggest an underlying role of aberrant immune responses to intestinal microbiota [36, 37]. IBD patients showed a decrease in microbial diversity, reduced abundance of several taxa in the* Firmicutes* phylum, and increased* Gammaproteobacteria* abundance [38, 39]. However, it is unclear whether these differences are a cause or consequence of IBD development.

Using FMT for ulcerative colitis (UC) treatment dates back to 1988, when the first idiopathic UC patient received treatment with FMT and was cured [40]. Furthermore, in a separate study, 6 relapsing UC patients experienced complete clinical, colonoscopic, and histological improvement after FMT [41]. Meta-analyses of FMT for IBD patients performed by Anderson et al. [42] showed that 63% of UC patients achieved remission, 76% could stop taking medications for IBD, and 76% experienced a decrease in GI symptoms. In a double-blinded RCT of FMT in active UC case, Moayyedi et al. [43] reported that 9 patients treated with FMT (24%) and 2 treated with placebo (5%) achieved remission at 7 weeks. Additionally, a recent randomized, double-blinded, placebo-controlled trial of multidonor, intensive-dosing FMT in patients with active UC [44] confirmed the primary outcome (steroid-free, clinical remission with endoscopic remission or response) was achieved after 8 weeks in 11 (27%) of 41 patients allocated to FMT versus 3 (8%) of 40 participants assigned to the placebo group (p=0.021). In another single-center, double-blinded, randomized, proof-of-concept clinical trial, Rossen et al. [45] suggested that, in the intention-to-treat analysis, 7 of 23 patients who were treated with FMT from healthy donors (30.4%) as well as 5 of 25 controls (20.0%) achieved the primary endpoint (p=0.51) in per protocol analysis, and 7 of 17 patients who received fecal transplants from healthy donors (41.2%) and 5 of 20 controls (25.0%) achieved the primary endpoint (p=0.29). In the phase 2 trials [45], there were no statistically significant differences in both clinical and endoscopic remission between UC patients who were treated with fecal microbiota from healthy donors or their own fecal microbiota. Thus far, it is difficult to make robust conclusions about the FMT's efficacy and safety for IBD due to a lack of uniformity in the therapy protocols and delivery approaches used in each study. The patient populations assessed in each study varied with respect to disease type, severity, phenotype, and concomitant medications. Additionally, although the donors were screened, they were not otherwise standardized or well characterized [46].

Borody et al. [47] suggested Crohn's disease (CD) is less effective to FMT than UC. Nonetheless, several case reports have demonstrated FMT as a promising treatment option for CD [48–50]. He et al. [51] suggested that sequential fresh FMT might be a strong treatment option to induce and maintain clinical remission in patients with CD complicated by an intraabdominal inflammatory mass. CD patients could be treated with a second FMT less than 4 months after the first course for maintaining beneficial effects [52]. After 1 month following FMT in CD patients, only 13.6% of mild adverse events occurred, including increased frequency of defecation, fever, abdominal pain, flatulence, hematochezia, vomiturition, bloating, and herpes zoster. No adverse events beyond 1 month were observed [53].

### 2.3. Irritable Bowel Syndrome (IBS)

Many studies have suggested that gut microbial alterations (reduced biodiversity and abundance of* Bacteroidetes*) are associated with IBS subsets [54, 55]. Germ-free mice treated with fecal transplants from diarrheal IBS (IBS-D) patients with or without anxiety experienced more rapid gastrointestinal transit, gut barrier dysfunction, anxiety-like behavior, and innate immune activation compared to mice treated with fecal transplants from healthy controls [56]. Holvoet et al. [57] conducted FMT in 12 patients with refractory IBS (Rome III criteria) experiencing intermittent diarrhea and severe bloating to find that 9 patients (75%) achieved the primary endpoint, 12 weeks after FMT. Responders were continually monitored to find that 7/9 (78%) still achieved IBS symptom relief after 1 year, suggesting a long-lasting efficacy of FMT. These results support promising microbiota-targeted therapies in IBS patients. A pilot study reported by Ge et al. [58] confirmed that FMT combined with fiber could also improve constipation in IBS patients by regulating gut microbiota. However, some studies offered different voices [59]. In a randomised double-blinded placebo-controlled study [60], FMT changed gut microbiota in patients with IBS, but patients in the placebo group experienced greater symptom relief compared with the FMT group. Therefore, a deeper understanding of the altered microbiota of patients with IBS and more rigorous trials are warranted before the utility of FMT for IBS.

## 3. FMT for Extraintestinal Disorders

### 3.1. Liver Disease

Changes in the intestinal microbiota are important for determining the occurrence and progression of chronic liver disorders such as alcoholic liver disease (ALD) [61–64], nonalcoholic fatty liver disease (NAFLD) [65–67], nonalcoholic steatohepatitis (NASH) [68–70], cirrhosis [71–73], and hepatocellular carcinoma (HCC) [74]. Research from a Chinese cohort in an open-label and single-blinded trial demonstrated that FMT could induce HBeAg clearance in a significant proportion of the cases with persistent positive HBeAg even after long-term antiviral treatment [75]. Ferrere et al. [76] found ALD was prevented in mice treated with alcohol-induced liver lesions by fecal transplantation from alcohol-fed mice resistant to ALD or with prebiotic (pectin).

Le Roy et al. [77] generated a mouse model to address the role of gut microbial communities in NAFLD development. The authors divided the conventional mice into responder and nonresponder groups, according to their response to high-fat diet (HFD), and showed that germ-free mice treated with FMT from different donors (responder or nonresponder) developed comparable results to the HFD group. The germ-free group treated with fecal transplants from the responders addressed steatosis and harbored larger abundance of* Roseburia* and* Barnesiella*. The content of* Allobaculum* was increased in the other group.

Hepatic encephalopathy (HE) is a decline in brain function that occurs as a result of severe liver disease. Gut microbial dysbiosis could be linked to minimal hepatic encephalopathy (MHE) in cirrhotic patients, especially with the ammonia-increasing phenotype in MHE. The intestinal urease-containing* Streptococcus salivarius* was absent in control group but present in cirrhotic patients with and without MHE.* Streptococcus salivarius* could be a promising target in cirrhotic patients with MHE [78]. Recurrent HE is common in cirrhotic patients despite the standard of care and may lead to irreversible neurocognitive injury [79]. HE patients have gut microbiota dysbiosis, which is partially driven by frequent antibiotic use, resulting in further HE recurrence [80]. Bajaj et al. [81] conducted an open-label, randomized clinical trial with a 5-month follow-up in outpatient cirrhotic men diagnosed with recurrent HE and found that FMT could reduce hospitalization and improve cognition as well as microbial dysbiosis in these patients.

### 3.2. Metabolic Diseases

Ridaura et al. [20, 21] demonstrated that gut microbial communities from obese or lean individuals induced similar phenotypes in mice and, more remarkably, that the microbiota from lean donors could invade and reduce adiposity gain in obese recipient mice. Fisher et al. [82] found no clinically relevant changes in recipient BMIs following a single FMT among patients with CDI, regardless of the donor BMI, within 12 months after FMT. FMT has also been tested in insulin resistance. Overweight patients with metabolic syndrome received microbiota from either their own feces (autologous transfer) or from lean healthy controls (allogeneic transfer). After 6 weeks, the allogeneic fecal transfer group had improved hepatic and peripheral insulin sensitivity by 119% and 176%, respectively, as shown using a euglycemic-hyperinsulinemic clamp technique [83].

Tang et al. [84], who performed two prospective clinical studies enrolling 4007 participants, as well as Wang et al. [85], who designed a cohort of 1876 subjects, found that the production of trimethylamine oxide (TMAO) from dietary phosphatidylcholine is dependent on metabolism by gut microbial communities and that increased levels of the microbial metabolite TMAO are associated with an elevated risk of incident major adverse cardiovascular events. In addition, TMAO increases risk of platelet hyperreactivity and thrombosis, and microbial transplantation suggests thrombosis is a transmissible trait [86]. Subsequently, Wang et al. [87] further discovered that a nonlethal inhibition of intestinal microbial trimethylamine production can be used to treat atherosclerosis.

Studies have also indicated that gut microbial dysbiosis is associated with type 2 diabetes (T2D) [88, 89]. The abundance of bacterial genera producing butyrate was found to be lower in metformin-untreated T2D patients compared to nondiabetic controls. Conversely, the increase in* Lactobacillus* previously observed in patients with T2D, without accounting for the treatment regimen, was eliminated when controlling for metformin treatment [88]. Wu et al. [90] conducted a placebo-controlled, randomized, double-blind study in individuals with newly diagnosed T2D who received metformin or placebo for 4 months and found that metformin had a strong impact on intestinal microbiota. They then transferred human fecal microbiota to germ-free mice in order to explore the role of metformin-altered microbiota on host glucose metabolism. They confirmed that altered gut microbiota could mediate the antidiabetic effects of metformin.

### 3.3. Chronic Kidney Disease (CKD)

Studies using 16S sequencing and microarray method have been initiated to explore the microbiota-kidney disorder axis. Significant differences in the microbiota composition were discovered in end-stage renal disease (ESRD) patients compared with healthy controls [91]. To investigate the effect of uremia on the microbiota, differences in the gut microbiota composition between ESRD patients and healthy individuals have been delineated [92]. ESRD patients exhibit an enriched microbiota with urease and uricase enzymatic activities, which could contribute to the elevated metabolism of urea linked with CKD. In contrast, Barros et al. [93] discovered no significant differences in the intestinal microbial profiles between a small cohort of CKD patients and healthy individuals. Indoxyl sulfate (IS) is a toxin that increases in plasma when the function of the kidneys declines, contributing to CKD progression [94–97]. Devlin et al. [98] identified a widely distributed family of indole-producing tryptophanases in commensal intestinal microbiota. They then engineered bacteria to control the* in vivo* production of the downstream product, the uremic toxin (IS). These results support a new option for CKD treatment by directing microbiota. Although this approach is far from clinical applications, future studies are needed to determine whether IS or other uremic solutes are true uremic toxins and potential therapeutic targets or simply biomarkers of advanced CKD [99, 100].

### 3.4. Neuropsychiatric Disorders

The intestinal microbiome plays major roles in immune, neuroendocrine, and neural pathways [101]. The brain-gut-microbiota axis is one of the most important pathways, whereas the gut microbiome can recruit bidirectional communication network to regulate the brain function, development, and even behavior [22, 102]. Experimental and clinical investigations underscore the important role of the gut microbiome in stroke pathogenesis [103, 104]. Based on these insights, targeting the intestinal microbiome is a potential treatment option for patients suffering from stroke [105].

Parkinson's disease (PD) is a progressive, chronic, and disabling neurodegenerative disease that begins in mid to late life. Li et al. [106] analyzed fecal microbial composition in 14 healthy volunteers and 24 PD patients using bacterial 16S rRNA sequencing. This study suggested that structural alterations in the intestinal microbiome in PD are characterized with reduced putative cellulose degraders and increased putative pathobionts. This could potentially decrease short-chain fatty acids (SCFAs) and produce more neurotoxins and endotoxins, which may be associated with the PD pathology development. In a previous study [107],* Blautia *was found to be markedly reduced in fecal samples and* Faecalibacterium* was decreased in colonic mucosal of PD patients. The first report in using FMT for PD treatment was from Austrian Professor Borody [108], who described a male PD patient suffering from chronic constipation where FMT eased the symptoms of PD. In a mouse model of PD [109], human *α*-synuclein protein is expressed at high levels in mice brains. These mice have disease characteristics including movement abnormalities, *α*-synuclein aggregation in neurons expressing the neurotransmitter dopamine, an immune response in the brain that includes the microglial cells activation, and the production of potentially neurotoxic cytokine molecules. When Sampson et al. [110] removed the intestinal microbiota from mice, the severity of disease symptoms was reduced. If PD mice lacking gut bacteria received FMT from diseased people, mice developed movement abnormalities that did not occur when fecal bacteria from healthy individuals were transplanted instead. In addition, using wild-type mice for the same transplant experiments did not result in movement abnormalities [111].

Alzheimer's disease (AD) is a severe and increasing socioeconomic burden. Harach et al. [112] showed a remarkable alteration in the fecal microbiota from an A*β* precursor protein (APP) transgenic AD mice model as compared to nontransgenic wild-type group. Colonization of germ-free APP transgenic mice with gut microbiome from conventionally raised APP transgenic animal elevated the cerebral A*β* pathology, while microbiota colonization from wild-type mice was less responsive for elevating cerebral A*β* levels.

Epilepsy contributes to seizure-related disability, mortality, comorbidities, stigma, and increased costs [113]. Recently, He et al. [114] reported the first case using FMT in seizure-related disability. This study found that FMT led to intestinal and neurological symptom remission in a girl with CD and a 17-year history of epilepsy. During a 20-month follow-up, FMT proved its effectiveness on preventing the relapse of seizures after withdrawal of antiepileptic medications.

Autism spectrum disorders (ASDs) are neurodevelopmental conditions, characterized by social and behavioral impairments. Wang et al. [115] analyzed 38 studies, including 25 animal studies and 15 human reports (2 studies were conducted in both), and concluded that probiotics [*Bifidobacterium* (e.g.,* B. breve*,* B. infantis*, and* B. longum*) and* Lactobacillus* (e.g.,* L. rhamnosus* and* L. helveticus*)] showed efficacy for easing psychiatric disorder-related behaviors such as anxiety, depression, ASD, obsessive-compulsive disorder, and memory abilities. Several reports have disclosed an aberrant gut microbiota in ASD [116–120]. There is report of autistic symptom remission in two children after FMT [121]. In a small open-label clinical trial with 18 ASD-diagnosed children, Kang et al. [122] suggested that FMT could alter the gut microbiota by increasing bacterial diversity and improving both gastrointestinal and autism symptoms. Parallel results have also been presented in an ASD mouse model, in which* Bifidobacterium fragilis* could improve anxiety-like behavior, sensory gating, and communicative behavior [17].

Depression is a common and heterogeneous disorder responsible for significant disability. Kelly et al. [123] recruited 34 depressed patients and 33 matched healthy individuals and confirmed that depression is associated with a decrease in intestinal microbiota abundance and biodiversity. FMT from patients with depression to microbiota-depleted rats could induce behavioral and physiological features characteristic of depression in the recipient rats, including anhedonia and anxiety-like behaviors, as well as alterations in tryptophan metabolism.

There is also emerging evidence showing that the intestinal commensal microbiome has an important role in the pathogenesis of multiple sclerosis (MS) [124–127]. Three MS patients treated with FMT for constipation eventually experienced both normal defecation and complete normalization of neurological symptoms, improving their life quality [124]. Borody et al. [128] presented a case report of a young woman with myoclonic dystonia and chronic diarrhea. These symptoms had codeveloped since she was 6 years old and gradually developed in severity. FMT resulted in improvements in diarrhea, myoclonus dystonia, and an improved ability to perform tasks requiring dexterity such as holding a cup and fastening buttons

Myalgic encephalomyelitis/chronic fatigue syndrome (ME/CFS), characterized by unexplained persistent fatigue, is commonly accompanied by sleeping disturbances, cognitive dysfunction, fever, orthostatic intolerance, lymphadenopathy, and IBS. Alterations in intestinal microbiota have also been explored in CFS patients [129]. The population of* E.coli* was decreased in CFS patients compared to healthy controls (49%* vs* 92.3%). ME/CFS is associated with microbial dysbiosis and distinct bacterial metabolic disturbances that may influence disease severity [130]. A recent study performed using a larger cohort with 60 CFS patients experiencing gastrointestinal symptoms who had undergone FMT [131] showed that 42/60 (70%) patients responded to FMT and 7/12 (58%) achieved a complete symptoms resolution after a 15-20-year follow-up. These results indicate that FMT could be used in the treatment of CFS.

### 3.5. Autoimmune Diseases

There are many publications indicating a relationship between intestinal microbiota alterations and autoimmune disorders including idiopathic thrombocytopenic purpura (ITP), systemic lupus erythematosus (SLE), arthritis, Sjogren's syndrome, and Hashimoto's thyroiditis [132]. In a case of UC with comorbid ITP, ITP symptoms have been shown to disappear, and platelet levels have been normalized after treatment with FMT [132]. While there is ample evidence [133, 134] indicating a relationship between the immune system and microbiota, a role for gut microbial dysbiosis in autoimmune disorders would not be surprising.

### 3.6. Allergic Disorders

Information about using FMT in allergic disorders such as food allergies and allergic asthma has not yet been reported. However, there is strong evidence suggesting that gut microbiome dysbiosis plays an important role in the etiopathogenesis of these disorders [135, 136]. The application of FMT appears to be promising and valuable for restoring immune homeostasis by transferring a complex bacteria community that is stable and easy to colonize [137].

### 3.7. Hematological Diseases

Studies have demonstrated that the gut microbiome has an impact on hematopoiesis [138, 139]. Antibiotics impair murine hematopoiesis by depleting the gut microbiota [140]. Furthermore, acute myeloid leukemia (AML) patients, presenting a high degree of intrapatient temporal instability of biodiversity, showed increased variability associated with adverse clinical outcomes [141]. Allogeneic stem cell transplantation (alloSCT) is one curative therapy for most hematologic malignancies. The success of this treatment is limited due to major complications, including graft-versus-host disease (GVHD). Varelias et al. [142] showed that recipient-derived IL-17A is critical for the intestinal acute GVHD prevention and that elevated susceptibility to acute GVHD could be transferred to wild-type mice via cohousing with IL-17RA- or IL-17RC-deficient mice.

### 3.8. Tumors and Gut Microbiota

A strong link has been demonstrated between the gut microbiome and cancer. Such examples are the links between* Fusobacterium nucleatum* and colorectal cancer [24, 143] or* Helicobacter hepaticus* in hepatocarcinogenesis [144]. Chemoimmunotherapy enhances antitumor effects via the synergism of chemotherapy and immunotherapy [145, 146]. Gut microbes have ascended to prominence as key modulators of host immunity, raising the possibility that they could influence the treatment outcome of cancer immunotherapy. Daillere et al. [147] showed that the antitumoral efficacy of cyclophosphamide (CTX) relies on two gut commensal species,* Enterococcus hirae* and* Barnesiella intestinihominis.* These bacteria alter the tumor microenvironment by reducing regulatory T cells and stimulating cognate antitumor cytotoxic T cell (CTL) responses. Vetizou et al. [148] found that the CTLA-4 blockade antitumor effects depended on distinct* Bacteroides species*. In both mice and patients T cell responses specific for* Bacteroides thetaiotaomicron* or* Bacteroides fragilis* were markedly linked to the efficacy of CTLA-4 blockade. Tumors with antibiotic-treated or germ-free mice did not respond to CTLA blockade. This defect was overcome by immunization with* Bacteroides fragilis* polysaccharides, or by adoptive transfer of* Bacteroides fragilis*-specific T cells. FMT from humans to mice further suggested that the treatment of melanoma patients with antibodies against CTLA-4 favored the outgrowth of* Bacteroides fragilis* with anticancer properties. Sivan et al. [149] also found that* Bifidobacterium* was associated with antitumor effects. Oral administration of* Bifidobacterium* alone could improve tumor control to the same degree as anti-PD-L1 therapy (checkpoint blockade), and combination treatment nearly abolished tumor growth. Recently, Wang et al. [150] reported that immune checkpoint inhibitors- (ICI-) associated colitis successfully treated along with FMT reconstituted the gut microbiome and increased colonic mucosa-related regulatory T-cells. These findings indicate that manipulating the gut microbiota may modulate cancer immunotherapy.

Radiation exposure in a mass casualty setting is a serious military and public health concern [151]. Exposure to a high dose of irradiation even in a short time can result in both gastrointestinal and bone marrow toxicities, which are considered as acute radiation syndrome (ARS) [152]. Cui et al. [153] discovered that the composition of gut microbiota differed between female and male mice and was also associated with susceptibility to radiation toxicity. They further showed that FMT could increase the survival rate in irradiated mice, increase peripheral white blood cell counts, and also improve gut function and gut epithelial integrity in irradiated animals. FMT might be a treatment strategy to reduce radiation-related toxicity and improve prognosis after radiotherapy.

## 4. Conclusions

FMT has become a well-established procedure and the most effective treatment option for recurrent CDI. Beyond the treatment of CDI, increasing studies have shown that FMT also presents potential and promising clinical indications for the treatment of many other disorders related to gut microbial dysbiosis. Additionally, well-designed, high-quality RCT researches are urgently needed to further identify the FMT's efficacy and safety for both inter- or extraintestinal disorders. It is expected that the FMT standardization, including donor selection, FMT material preparation, and administration routes, will soon be established and its applications expanded. Therefore, it is of great value to elucidate the effects of FMT as a promising and alternative treatment for some other diseases related to the intestinal microbiome.

## Figures and Tables

**Figure 1:**